# Tests of human auditory temporal resolution: preliminary investigation of ZEST parameters for amplitude modulation detection

**DOI:** 10.3389/fnins.2023.1148476

**Published:** 2023-07-05

**Authors:** Shuji Mori, Takashi Morimoto, Yuto Murata, Yasuhide Okamoto, Sho Kanzaki

**Affiliations:** ^1^Department of Informatics, Faculty of Information Science and Electrical Engineering, Kyushu University, Fukuoka, Japan; ^2^Technical Development Center, RION Co., Ltd., Tokyo, Japan; ^3^Department of Informatics, Graduate School of Information Science and Electrical Engineering, Kyushu University, Fukuoka, Japan; ^4^Department of Otorhinolaryngology, Tokyo Saiseikai Central Hospital, Tokyo, Japan; ^5^Keio University School of Medicine, Tokyo, Japan; ^6^Division of Hearing and Balance Research, National Institute of Sensory Organs, National Hospital Organization Tokyo Medical Center, Tokyo, Japan

**Keywords:** auditory temporal resolution, amplitude modulation, threshold estimation, ZEST, simulation

## Abstract

Auditory temporal resolution plays a critical role in the everyday experience of listening to complex acoustic patterns. Amplitude modulation detection thresholds are widely used to measure auditory temporal resolution. In an attempt to develop a standardized clinical test of auditory temporal resolution, we used ZEST (Zippy Estimation by Sequential Testing, a Bayesian threshold estimation procedure, to measure amplitude modulation detection thresholds. ZEST utilizes prior knowledge about a listener’s thresholds, as represented by a probability density function of the thresholds, and psychometric functions of the listener’s responses. This paper reports a preliminary study in which ZEST parameters that could be used for measurements of amplitude modulation detection thresholds were sought. For this purpose, we created histograms of the detection thresholds for a wide range of modulation frequencies, measured the psychometric functions of amplitude modulation detection, and performed computer simulations of ZEST threshold estimation. The results suggested that, with appropriately-set parameters, ZEST allows for the accurate estimation of amplitude modulation detection thresholds within 20 trials.

## 1. Introduction

Auditory temporal resolution, the ability to detect temporal changes in sounds, is vital for the everyday activity of listening to complex acoustic patterns. Auditory signals are represented in the time and frequency dimensions, and they contain rich information in their temporal envelopes which are detected, discriminated, and interpreted by the human auditory system. This is particularly true in speech perception. Speech signals can be categorized into different phonemes for a given language on the basis of differences in temporal structures, which can be as small as several milliseconds (Plack, 2005). It has been suggested that deficits in temporal resolution are linked to difficulties in understanding speech, particularly in noisy environments (Plack et al., 2014; Oxenham, 2016).

Amplitude modulation (AM) detection offers a way to assess one aspect of a listener’s auditory temporal resolution. In a widely-used AM detection method (e.g., Bacon and Viemeister, 1985; Shen and Richards, 2012; Morimoto et al., 2018), the signals are sinusoidally modulated on a noise carrier, and the minimum detectable depth of modulation, or AM detection threshold, is measured and used to construct a temporal modulation transfer function (TMTF), which relates the AM detection threshold to the modulation frequency (*f*_m_). When a broadband noise carrier is used, TMTF has been found to exhibit low-pass characteristics, with low thresholds seen at low *f*_m_, which gradually increase for higher *f*_m_ (Viemeister, 1979; Eddins and Green, 1995). The flat portion of the TMTF curve is termed peak sensitivity (*S*_p_), with the cut-off frequency denoted as *f*_m_. These two parameters can be regarded as indices of auditory temporal resolution. In listeners with normal hearing (NH), *S*_p_ ranges from −20 to −25 dB (transformed from the modulation depth, m , by $20\log_{10}m$ ), and *f*_c_ varies from 32 to 150 Hz, depending on the intensity and spectral features of the carrier used (Viemeister, 1979; Eddins and Green, 1995; Strickland and Viemeister, 1997). When compared with NH counterparts in the same experiments, hearing-impaired (HI) listeners showed higher *S*_p_ and lower *f*_c_ values, indicating degradation of their temporal resolution (Bacon and Viemeister, 1985; Morimoto et al., 2018). Notably, Morimoto et al. (2018) found that some HI listeners were unable to perform AM detection at high *f*_m_, which would typically be easily accomplished by NH listeners.

One obstacle to obtaining *S*_p_ and *f*_c_, particularly in the clinical setting, is the total measurement time required since it is necessary to obtain measurements at multiple *f*_m_ to construct the TMTF curve. To counter this problem, a couple of efficient procedures for estimating *f*_c_ and *S*_p_ from only two measurement points have been proposed (Shen and Richards, 2012; Morimoto et al., 2018). Both of these procedures are based on the assumption that the TMTF can be approximated by a first-order low-pass filter function, so the exact shape of the function can be estimated from only two points, one at a constant threshold level for low *f*_m_ and the other along the cut-off portion at high *f*_m_. Shen and Richards (2012) and Morimoto et al. (2018) showed that the estimates of *f*_c_ and *S*_p_ obtained from their procedures were in general agreement with those obtained using the conventional method of measuring thresholds at multiple *f*_m_.

While such two-point procedures appear to be promising in terms of their ability to reduce the measurement time, two issues remain. First, measurement errors at the two points can potentially result in large estimation errors of *S*_p_ and *f*_c_. Shen and Richards (2012) reported that in one of their three participants their two-point procedure yielded a much higher threshold than the conventional method, resulting in considerable error in the estimates of *S*_p_ and *f*_c_ between the two methods. Second, the reduction in the measurement time is limited by the threshold estimation method used. Shen and Richards (2012) and Morimoto et al. (2018) both used a 1-up 2-down method to measure the AM detection threshold and the just-noticeable value for *f*_m_. Morimoto et al. (2018) noted that the 1-up 2-down method was time-consuming, as over 50 trials were required.

Due to these two issues, we took a different approach to reducing the TMTF measurement time. We aimed to reduce the number of trials needed to measure AM detection thresholds. For this purpose, we used ZEST (Zippy Estimation by Sequential Testing; King-Smith et al., 1994), an adaptive Bayesian threshold estimation procedure. ZEST was originally developed for estimating thresholds for visual contrast detection (King-Smith et al., 1994) and has been successfully applied to automated static perimetry for detecting glaucoma-related visual field loss (Vingrys and Pianta, 1999; Turpin et al., 2003). In addition, ZEST was used for measuring thresholds for intensity discrimination (Marvit et al., 2003). As far as we know, ZEST has never been used for AM detection.

ZEST has been found to be one of the most efficient adaptive threshold estimation methods (Treutwein, 1995; Turpin et al., 2002; Marvit et al., 2003). When the relevant parameters are properly set, ZEST requires about 10 trials to obtain a reliable threshold measurement (Marvit et al., 2003; Turpin et al., 2003). Such a small number of trials should reduce the total TMTF measurement time, even if the TMTF curve is constructed from multiple thresholds over a wide range of *f*_m_.

To establish reliable and efficient methods of threshold estimation, computer simulations and the subsequent applications of the results to human participants are necessary (Treutwein, 1995). Computer simulations can be used to examine the performance of a threshold estimation method by comparing siulated estimates of thresholds with pre-specified values of true thresholds. The methodologies and parameters suggested by the computer simulations can then be applied to human participants to validate them. This paper reports the results of simulations of ZEST as a tool for AM detection threshold estimation.

In the following sections, we first explain ZEST, its assumptions, parameters, and measurement processes. Next, we describe the set of initial parameters that were used in the computer simulations. We estimated these parameter values from our own dataset of thresholds and an experiment measuring psychometric functions for AM detection. Finally, we present the computer simulations, which were used to examine these parameters in terms of their efficiency and reliability for estimating AM detection thresholds at *f*_m_ values from 8 to 256 Hz.

## 2. ZEST

ZEST is a variant of QUEST (Watson and Pelli, 1983), which is also an adaptive threshold measurement procedure based on Bayes’ theorem. ZEST (and QUEST) make three assumptions: (1) psychometric functions have the same shape along a log scale of stimulus values, (2) the observer’s threshold does not change during the measurement period, and (3) individual trials are statistically independent. During the measurement period, ZEST updates the probability density function (*pdf*) of the target threshold based on prior knowledge of the *pdf* and the observer’s response. This process is expressed by the following equation (King-Smith et al., 1994):


(1) $$q_{i}\left(T\right)=p\left(r_{i},x_{i},T\right)q_{i-1}\left(T\right)$$

where $q_{i}\left(T\right)$ is the *pdf* of the threshold in trial i [based on the assumption (1), the stimulus values are all log-transformed], and $p\left(r_{i},x_{i},T\right)$ is the probability of the observer making response $r_{i}$ (1 for “yes” or correct, 0 for “no” or incorrect) to the presented stimulus, $x_{i}$ , given that the observer’s threshold is T . When $r_{i}=1$ , $p\left(r_{i},x_{i},T\right)$ is given by the psychometric function, Ψ(X) :


(2) $$p\left(1,x_{i},T\right)=\Psi \left(x-T\right)$$

When $r_{i}=0$ , $p\left(0,x_{i},T\right)=1-p\left(1,x_{i},T\right)$ , so that


(3) $$p\left(0,x_{i},T\right)=1-\Psi \left(x-T\right)$$

In each trial, the stimulus value, $x_{i}$ , is set at the mean of $q_{i-1}\left(T\right)$ , the *pdf* obtained in trial i−1 [in QUEST, $x_{i}$ is set to the mode of $q_{i-1}\left(T\right)$ ]. Specifically, $q_{0}\left(T\right)$ is called the initial *pdf*, which represents knowledge about possible threshold values obtained prior to the measurement.

**Figure 1:**
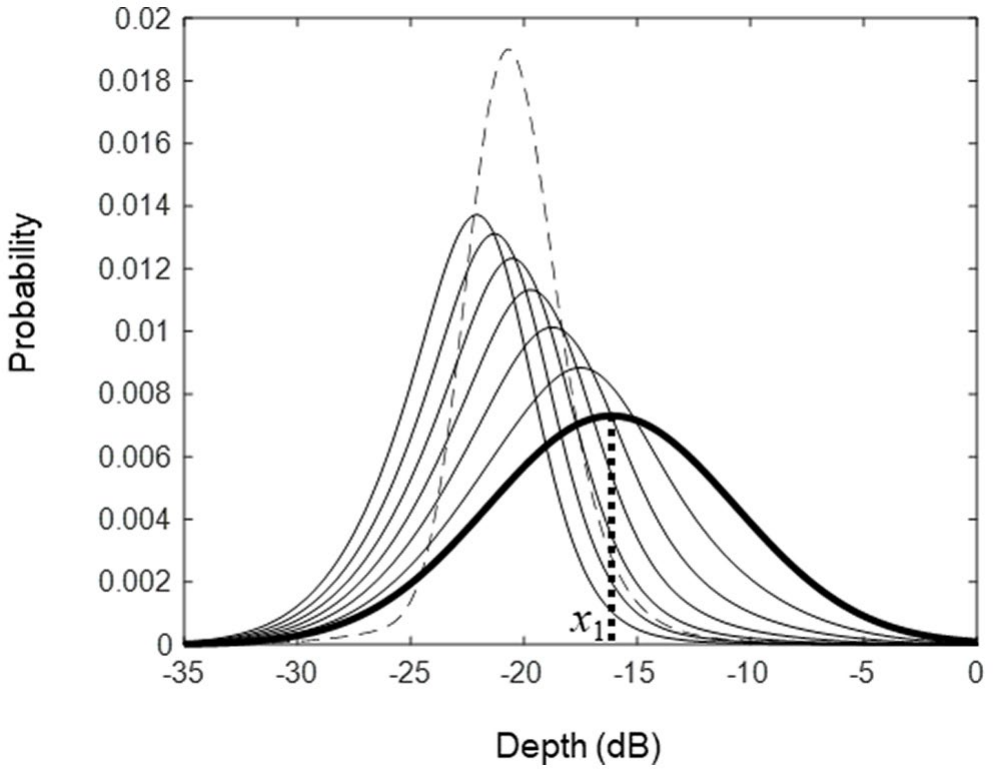
An illustration of the initial and posterior *pdfs* seen during the course of threshold estimation from trials 1 to 7. The rightmost distribution (thick line) shows the initial *pdf* and its mean was used as the stimulus in trial 1 ( $x_{i}$ ). The responses in trials 1 to 6 were correct, so in those trials, the posterior *pdfs* shifted to the left. In trial 7, the response was incorrect, and therefore, the posterior *pdf* (broken line) shifted to the right.

Figure 1 illustrates how the measurement progresses as the number of trials increases in ZEST. In trial 1, the stimulus $x_{1}$ is presented at the mean of the initial *pdf*, $q_{0}\left(T\right)$ . Since the response $r_{1}$ is correct ( =1 ), the posterior *pdf*, $q_{1}\left(T\right)$ is updated to a smaller mean than the mean of $q_{0}\left(T\right)$ . The posterior *pdf* moves to the left along a log stimulus scale when the response is correct and moves to the right when the response is incorrect (the broken-line distribution shown in Figure 1). Generally, we expect that the variance of the *pdf* will become smaller as the number of trials increases. The measurement process is terminated when the predetermined number of trials are completed, or when the variance of the *pdf* becomes smaller than a predetermined size. Either way, the mean of the posterior *pdf* in the final trial is taken as the threshold estimate.

The precision and efficiency of ZEST for estimating the observer’s threshold depends on the initial *pdf*, $q_{0}\left(T\right)$ , and the psychometric function, Ψ(X) , used for the measurement (King-Smith et al., 1994). ZEST is most efficient; i.e., requires fewest trials for threshold estimation, when its initial *pdf* approximates the threshold distribution of the target population, which can be inferred from a representative set of samples (Turpin et al., 2002, 2003). It is also noted that the performance of ZEST is relatively unaffected by deviations of the assumed initial *pdf* from the true one (King-Smith et al., 1994). The shape of a psychometric function is specific to the measurement procedure and condition. King-Smith et al. (1994) used a Weibull distribution for the psychometric function:


(4) $$\Psi \left(x-T\right)=1-\delta -\left(1-\gamma -\delta \right)\exp\left(-10^{\beta \left(x-T+\varepsilon \right)}\right)$$

where δ and γ are the false-negative and -positive rate, respectively; β is a shape parameter, which determines the slope of the psychometric function; and ε is a parameter for the sweat factor, which determines the detection probability of the thresholds. The false-positive rate, γ , is usually set at the chance level of the observer’s responses in the task used (e.g., 0.5 for a 2-interval forced-choice, 0.33 for a 3-interval forced-choice). Both the false-negative rate, δ , and the shape parameter determining the slope of the psychometric function, β , can be set from empirical data. The sweat factor, ε , will be specified when the other three parameters are set (see below).

## 3. Parameters for estimating AM detection thresholds

In this section, we describe the estimation of an initial *pdf* and a psychometric function, or more specifically the parameter values defining them, for AM detection. To the best of our knowledge, no previous studies have described the threshold distributions of AM detection. Regarding psychometric functions, the only related study we know of is Eddins (1993), who measured psychometric functions by a method of constant stimuli with a 2-interval forced-choice procedure, in which narrow-band noises were used as carriers with various modulation frequencies.

The initial *pdf* and psychometric function for AM detection may vary with the measurement method; i.e., the stimuli, task, and target populations. The method used for the subsequent applications of the obtained parameters to human measurements followed that of our previous studies (Mori et al., 2018; Morimoto et al., 2018). Briefly, the stimuli were sinusoidal AM sounds created using broadband noise as a carrier, with modulation frequencies from 8 to 256 Hz. The participants were NH or HI listeners, and the task was a 3-interval forced-choice task (for more details, see Mori et al., 2018, and Morimoto et al., 2018). Therefore, the psychometric functions measured by Eddins (1993) were not appropriate for estimating psychometric function parameters for our study. The following sections describe how we estimated the parameter values of the initial *pdf* and psychometric function specific to this measurement method.

### 3.1. Initial *pdf*

**Figure 2:**
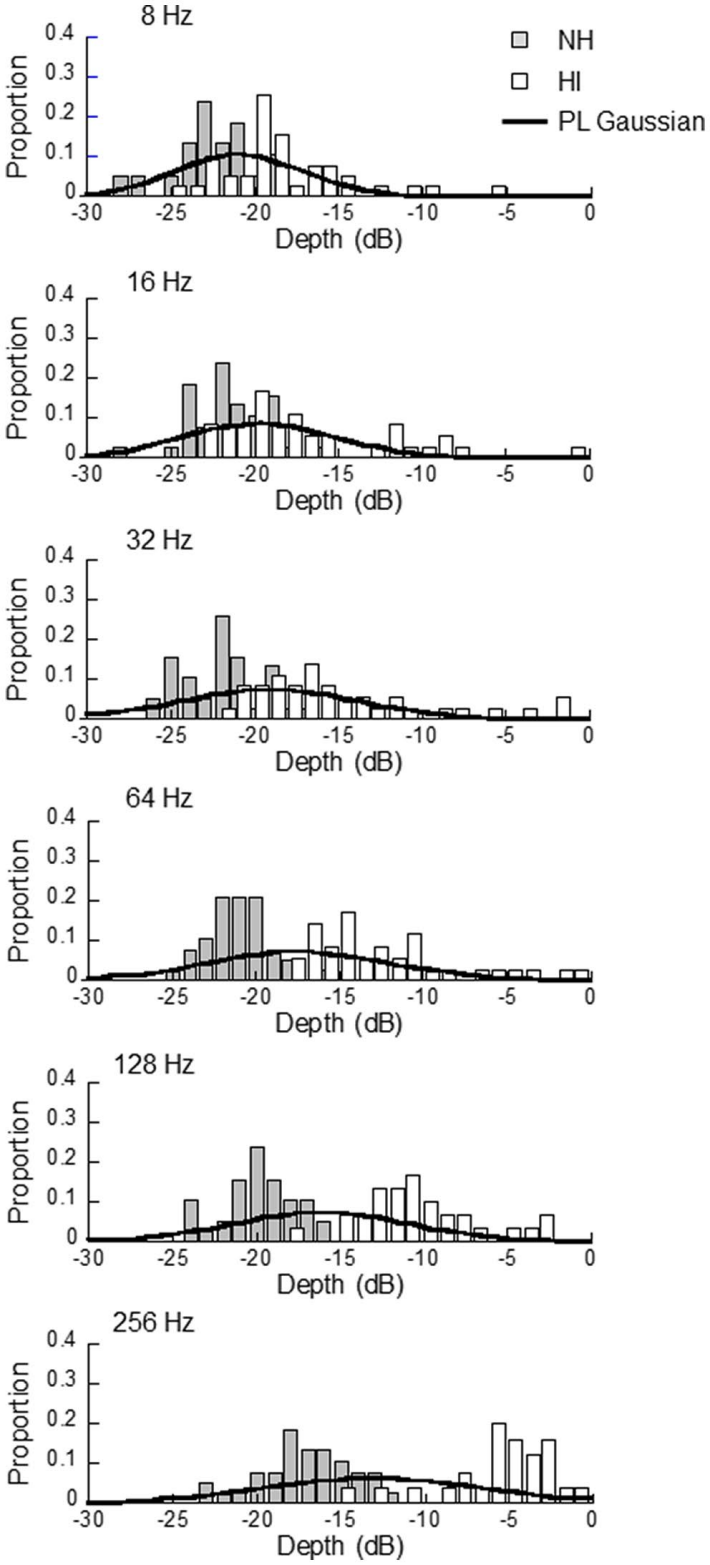
Histograms of threshold values obtained from the normal hearing (NH) and hearing impaired (HI) listeners. In the NI group, 38 thresholds were obtained at all modulation frequencies, while in the HI group the number of thresholds obtained was 39, 36, 36, 35, 30, and 26, respectively, for modulation frequencies of 8, 16, 32, 64, 128, and 256 Hz. The histograms of the HI group have been slightly shifted rightward to avoid concealing the overlapping bars of the NH group. The solid lines show Gaussian distributions with the mean and SD of the pooled data (PL) from the NH and HI groups.

It is often convenient to assume that the initial uncertainty in a threshold distribution is normally distributed. In this case, the threshold distribution of AM detection can be given by the following equation:


(5) $$q_{0}\left(x\right)=\left(1/\sqrt{2\pi \sigma_{0}^{2}}\right)\exp\left(-0.5\times {\left(\left(x-\mu_{0}\right)/\sigma_{0}\right)}^{2}\right)$$

with the mean, $\mu_{0}$ , and standard deviation (SD), $\sigma_{0}$ , estimated from the experimenter’s experience (Watson and Pelli, 1983; King-Smith et al., 1994). To estimate $\mu_{0}$ and $\sigma_{0}$ , we used our own dataset of thresholds, which consisted of the data collected by Morimoto et al. (2018) and Mori et al. (2018), and unpublished data, which were obtained using the same measurement method as that used in the latter two studies. There were a total of 38 NH participants and 39 HI participants.^1^ Figure 2 shows histograms of the thresholds for the NH and HI participants, separately for the modulation frequency, and Table 1 shows the means and SDs of the thresholds for the NH and HI groups, and the two groups pooled (hereafter referred to as the PL group). Gaussian distributions with the means and SD values of the thresholds of the PL group are also shown in Figure 2. As shown in the figure, the histograms for the NH and HI groups were partially overlapped but were separable from each other, and they shifted to the right (i.e., toward higher thresholds) as the modulation frequency increased. In the subsequent simulations, we examined the three sets of mean and SD values; i.e., those for the NH, HI, and PL groups, for the initial *pdf*, separately for the modulation frequency, to see which of these three sets of mean and SD values led to the best ZEST performance.

**Table 1:** Means (and standard deviations) of thresholds of the normal hearing (NH), hearing impaired (HI), and pooled data (PL) groups.

	Modulation frequency (Hz)					
	8	16	32	64	128	256
NH	−22.99 (2.35)	−21.97 (2.28)	−22.50 (2.37)	−21.44 (1.89)	−20.25 (2.13)	−17.56 (2.73)
HI	−19.03 (3.95)	−17.22 (5.19)	−15.55 (5.35)	−12.89 (4.56)	−10.92 (3.59)	−6.22 (3.29)
PL	−20.98 (3.80)	−19.66 (4.61)	−19.12 (5.36)	−17.34 (5.49)	−16.13 (5.46)	−13.06 (6.32)

There were 38 participants in the NH group, whereas the number of participants who underwent testing at each modulation frequency varied in the HI group (39, 36, 36, 35, 30, and 26 participants, respectively, at 8, 16, 32, 64, 128, and 256 Hz).

### 3.2. Psychometric function

To estimate the psychometric function parameters, specifically β and δ from Equation 4, we conducted an experiment with similar stimuli and a similar task to those used by Mori et al. (2018) and Morimoto et al. (2018).

#### 3.2.1. Method

The stimuli were constructed from a broadband noise carrier (20 to 14,000 Hz) and had a duration of 500 msec (cos rise/fall 2.5 msec). The modulation frequency was either 8, 16, 32, 64, 128, or 256 Hz, with the modulation depth set at 5 levels for each frequency (see below). They were generated by a personal computer (FUJITSU, LIFEBOOK WA3/D1) using MATLAB software, which also controlled the stimulus presentation and data collection. The sampling rate was 48 kHz with a precision of 16 bits. All of the stimuli were presented to the participant’s left ear at 60 dB SPL through an audio interface (EDIROL, UA-3D) via headphones (SONY, MDR-27). Prior to the first experimental session of the day, sound pressure levels were calibrated by playing the individual signals to be used and recording their levels using a Brüel & Kjaer sound level meter (model 2250) with a 1/2-inch condenser microphone (model 4192) placed into an artificial ear (model 4153), which was used as an acoustic coupler.

The experiment was conducted in a sound-attenuated room. For each modulation frequency, the 70.7% detection threshold was first measured using the 1-up 2-down staircase method with a 3-interval forced-choice (3IFC) procedure. In each trial, a 1000-Hz tone was presented for 100 msec as a warning signal, followed by a 500-ms silent period and three 500-msec intervals (each separated by a 500-msec silent period). A modulated signal was presented at one of the three intervals, and an unmodulated signal was presented at the other two intervals. The participant’s task was to indicate the interval containing the modulated signal, by clicking on one of three boxes shown side-by-side on the computer monitor. The responses were self-paced, and correctness feedback was given by flashing the box corresponding to the correct response. The next trial immediately started after the feedback ended. The modulation depth was initially set at 0 dB and was changed by 4 dB for the first four reversals and 2 dB for the rest. Each measurement continued until 12 reversals, and the average of the last 8 reversals was taken as the threshold estimate for that run. The threshold measurement was repeated until the SD of all the estimates was below 2 dB. The mean of the threshold estimates was used as the middle of the 5 modulation depth levels used for the subsequent method of constant stimuli. The other 4 levels were set at ±3 and ± 6 dB of the middle level.

One of the 5 levels was presented in each trial in the method of constant stimuli, which was performed with a 3IFC procedure and started immediately after the threshold measurement. The response mode was the same as that used for the threshold measurement. The lowest and highest modulation depths were presented in 50 trials, and the other modulation depths were presented in 100 trials, and they were mixed randomly in a single session of 500 trials for each modulation frequency. The order of testing the five modulation frequencies was randomized across the participants. The participants took a rest between sessions, and they were allowed to take a rest at any time during a session.

**Figure 3:**
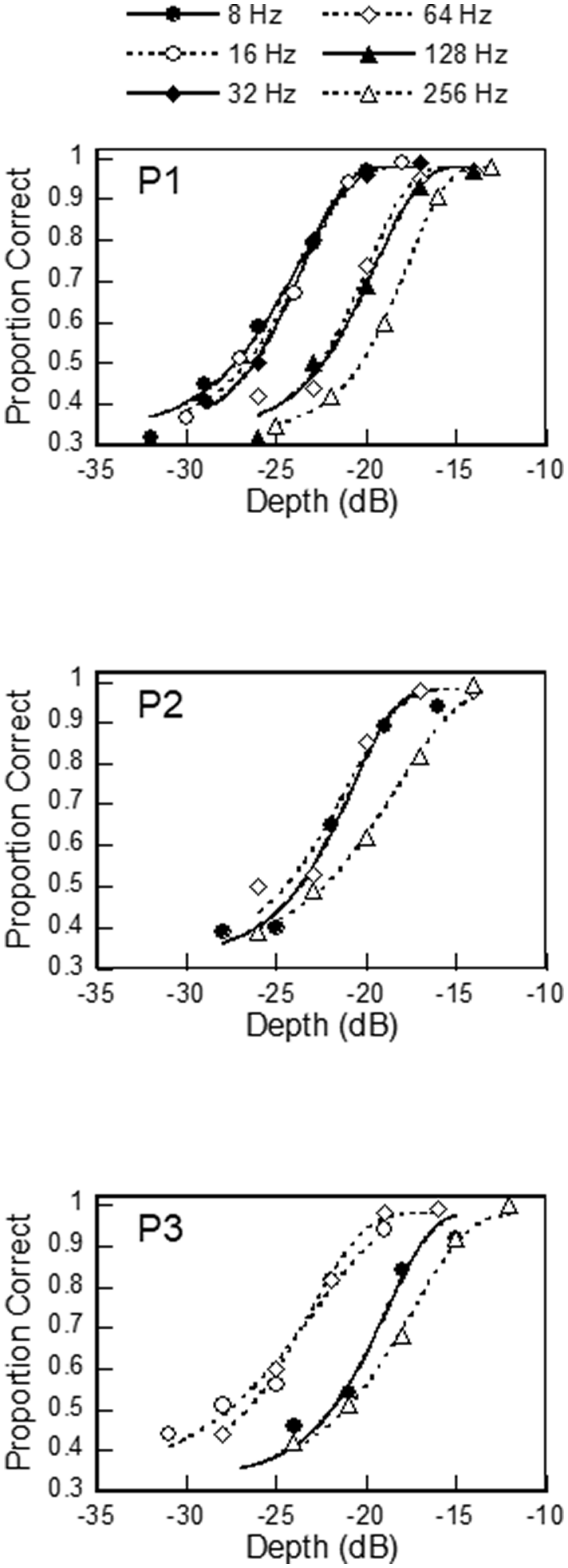
Psychometric functions obtained from 3 listeners. The curves were fitted with the Weibull distribution.

There were three participants, the first (P1) and second (P2) were authors (61 and 23 years old, respectively), and an undergraduate student (P3) (22 years old), who was naïve to the purpose of this study and gave his informed consent. All were men. P1 underwent testing at all of the modulation frequencies from 8 to 256 Hz, whereas P2 and P3 underwent testing involving some of these frequencies (see Figure 3 and Table 2). This experiment was approved by the Research Ethics Board of the Faculty of Information Science and Electrical Engineering, Kyushu University.

**Table 2:** Proportions of incorrect responses *P*(*i*) at the greatest modulation depth and the β values of the best-fit Weibull distributions for the three participants are shown in Figure 3.

		Modulation frequency (Hz)					
		8	16	32	64	128	256
P1	*P*(*i*)	0.03	0.01	0.01	0.03	0.03	0.02
	β	0.15	0.16	0.19	0.19	0.18	0.20
P2	*P*(*i*)	0.06			0.02		0.01
	β	0.18			0.15		0.13
P3	*P*(*i*)	0.08	0.06		0.01		0
	β	0.17	0.11		0.15		0.14

#### 3.2.2. Results

Figure 3 shows the psychometric functions of the three participants, which were constructed from their responses in the method of constant stimuli. The values of the psychometric functions ranged from 0.3 (chance level) to near 1.0 (certainty). The psychometric functions for low *f_m_* were located toward the lower modulation depth and generally shifted toward higher values as *f_m_* increased.

Table 2 shows the proportions of incorrect responses for the three participants at the highest modulation depth for each modulation frequency. These values are relevant to the setting of δ , the false-negative rate of the psychometric function, because the psychometric function is expected to reach a correct response rate of 1.0 as the modulation depth increases, and any incorrect (negative) responses at the highest modulation depth are taken as false negatives. Across the three participants, the proportions of incorrect responses ranged from 0.06 to 0, and there seemed to be no systematic difference in the incorrect response rate due to modulation frequency. Since most of the values were between 0.01 and 0.03, δ was set to 0.02 in our study. δ = 0.02 was also used by King-Smith et al. (1994) and Marvit et al. (2003) in their ZEST implementations.

Table 2 also shows the β values of the best-fit Weibull distributions (Equation 4) for the psychometric functions when the values of the other parameters were set as follows: γ = 0.33, δ = 0.02, and ε = 0. The best-fit distributions are also shown in Figure 3. The fits were quite good, with *R*^2^ higher than 0.98 for all of them. The β values ranged between 0.11 and 0.19 across the three participants. In P1, the β values increased with the modulation frequency, while in the other two participants there appeared to be no systematic trend relating to the modulation frequency. In the subsequent simulations, we manipulated the β value to see how it would affect the performance of ZEST.

The value of ε determines the detection probability of the threshold estimated by ZEST. ZEST is most efficient when ε is set to a value corresponding to the ideal sweat factor; i.e., the minimum value produced by the following equation:


(6) $$\frac{\Psi \left(x\right)\left[1-\Psi \left(x\right)\right]}{{\left[d\Psi \left(x\right)/dx\right]}^{2}}$$

(Taylor, 1971). As is clear in Equation 6, the ideal sweat factor, and the corresponding value of ε , depend on the form of the psychometric function. Since we fixed the values of γ and δ , the value of ε corresponding to the ideal sweat factor changed with the β value used. In the following simulations, ε was adjusted depending on the β value used, in order to obtain the most efficient estimates using ZEST. This adjustment set the detection probability of the estimated threshold at 0.84 for the Weibull distribution used in this study (Equation 8).

**Figure 4:**
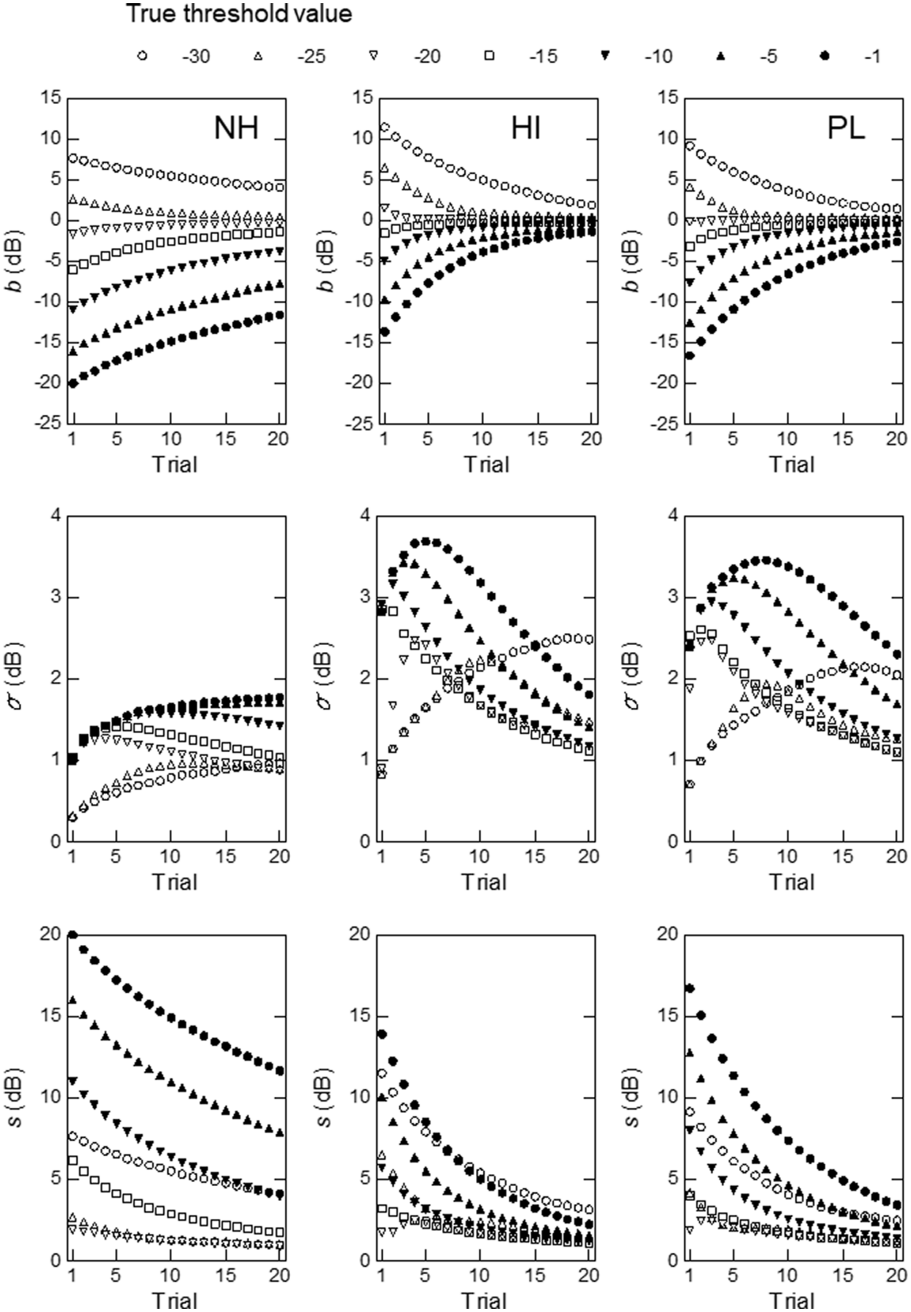
Simulation results obtained at a modulation frequency of 16 Hz with $\mu_{0}$ and $\sigma_{0}$ set at the mean and standard deviation of the thresholds for the NH (left), HI (middle), or PL (right) group. The measures used were, from top to bottom, the measurement bias, the standard deviation of the threshold estimates, and the total rms error.

## 4. Simulations

### 4.1. Method

Simulations were run on personal computers (DELL, OptiPlex 7070; ASUS, ZenBook UX305) using MATLAB (Mathworks, Inc.). Measurements for up to 20 trials were simulated for modulation frequencies of 8, 16, 32, 64, and 128 Hz, all with assumed true thresholds, T , ranging from −1 dB, or − 5 to −30 dB in 5-dB steps, which covered the range of potential threshold values for the NH and HI listeners (see Figure 1). All of the simulations were conducted using an exact enumeration technique (Mckee et al., 1985; King-Smith et al., 1994), in which all of the possible sequences of responses (either 1 or 0) from trial 1 to *N* were simulated, and the posterior *pdf*, $q_{Nj}\left(T\right)$ , for sequence *j* in trial *N* was computed for all sequences, and the mean of that *pdf*, $E_{j}$ , was taken as the threshold estimate for sequence *j*. The exact enumeration technique exhausts all possible threshold estimates and their probabilities of occurrence in trial *N*, resulting from the sequences of responses from the first trial to trial *N*. For example, when *N* = 1, there are only two sequences, either a correct (1) or an incorrect response (0) in that trial, yielding two threshold estimates. When *N* = 2, there are four possible sequences of responses from trial 1 to 2; i.e., 1 and 1, 1 and 0, 0 and 1, 0 and 0. These sequences yielded different threshold estimates because they were computed using Equation 1 and different prior *pdf*, $q_{i-1}\left(T\right)$ , and psychometric functions $p\left(ri,x_{i},T\right)$ depending on the responses in trials 1 and 2. There are 2*^N^* sequences for trial *N*; 2^3^ = 8 for *N* = 3, …2^20^ = 1048576 for *N* = 20. The exact enumeration technique has an advantage over the Monte Carlo technique in that the former provides more accurate statistical properties of sample distributions (McKee et al., 1985). This allowed us to evaluate the efficiency and precision of threshold estimates for various parameters of the initial *pdf* and psychometric functions.

For all of the simulations conducted in this study, the values of the parameters γ and δ were fixed at 0.33 and 0.02, respectively (see the *Psychometric function* section). The values of the other parameters; i.e., $\mu_{0}$ and $\sigma_{0}$ of the initial *pdf*, and β and ε of the psychometric function were manipulated to see their effects on the simulated performance of ZEST.

To evaluate the effects of these manipulated parameter values on the threshold estimate $E_{j}$ , we used three measures: measurement bias, the SD of $E_{j}$ , and the total root-mean-square (rms) error of $E_{j}$ relative to the true threshold (King-Smith et al., 1994). The measurement bias, b , is the difference between the expected value, $\hat{E}$ , of the threshold estimate in the *Nth* trial, and the true threshold T :


(7) $$b=\hat{E}-T$$

where


$$\hat{E}=\overset{2^{N}}{\underset{j=1}{\sum }}E_{j}p\left(E_{j}|T\right)$$

$$p\left(E_{j}|T\right)=\frac{q_{Nj}\left(T\right)}{q_{0}\left(T\right)}$$

The SD, σ , of the threshold estimates, $E_{j}$ , from their expected values, $\hat{E}$ , is given by:


(8) $$\sigma =\sqrt{\overset{2^{N}}{\underset{j=1}{\sum }}{\left(E_{j}-\hat{E}\right)}^{2}p\left(E_{j}|T\right)}$$

The total rms error, s , is given by:


(9) $$s=\sqrt{\overset{2^{N}}{\underset{j=1}{\sum }}{\left(E_{j}-T\right)}^{2}p\left(E_{j}|T\right)}=\sqrt{b^{2}+\sigma^{2}}$$

The efficiency can be evaluated from the measurement bias, assuming that the threshold estimates are likely to reach their true value eventually unless the parameter values used are unrelated to the underlying mechanism. The measurement bias, computed as a function of the trial, shows how quickly the threshold estimates approached the true threshold. The SD of the threshold estimates reflects the precision of these estimates; i.e., how variable they are. The total rms error is a composite index of both, as shown in Equation 9.

### 4.2. Results

#### 4.2.1. Effects of $\mu_{0}$ and $\sigma_{0}$

Simulations were conducted by setting the values of $\mu_{0}$ and $\sigma_{0}$ at the mean and SD values of the thresholds for the NH, HI, or PL group for each of the 5 modulation frequencies (Table 1). For these simulations, the values of β and ε were fixed at 0.18 and 1.0345, respectively. We obtained very similar results with other values of these parameters (see below).

**Figure 5:**
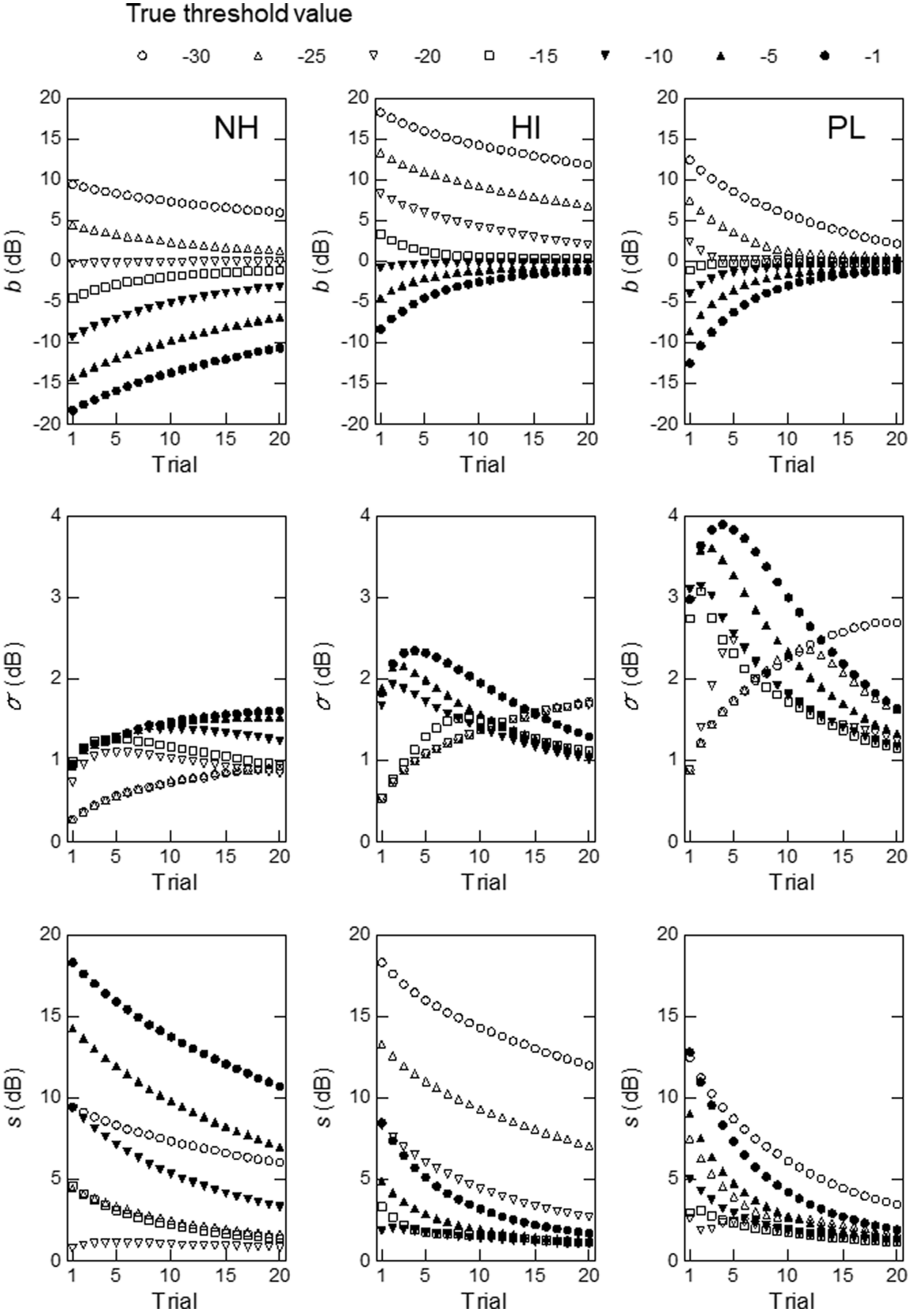
Simulation results obtained at a modulation frequency of 128 Hz with $\mu_{0}$ and $\sigma_{0}$ set at the mean and standard deviation of the thresholds for the NH (left), HI (middle), or PL (right) group. The measures used were, from top to bottom, the measurement bias, the standard deviation of the threshold estimates, and the total rms error.

Figures 4, 5 show the results obtained for modulation frequencies of 16 and 128 Hz, respectively (the results for the other modulation frequencies are shown in the Supplementary material). In the simulations shown in Figure 4, we begin by examining the top row of graphs, which illustrate the measurement bias b as a function of trials. b is the difference between the expected value of the threshold estimates at a given trial, $\hat{E}$ , and true threshold, T (Equation 7). $\hat{E}$ is computed from all possible threshold estimates, $E_{j}$ , and their associated probabilities of occurrence, $p\left(E_{j}|T\right)$ , in trial *N*. For a given initial *pdf* (in our case $\mu_{0}$ and $\sigma_{0}$ in Equation 5), b varies with the number of trials *N* and T . When the mean and SD values for the NH (left) group were used, b only approached 0 as the trial number increased for T = −25 to −15 dB. This result can probably be explained by the fact that the mean threshold for the NH group ( $\mu_{0}$ = −22.99 dB) lay close to these values of T . For other values of T , b started far from 0 and did not come close to 0 by trial 20.

**Figure 6:**
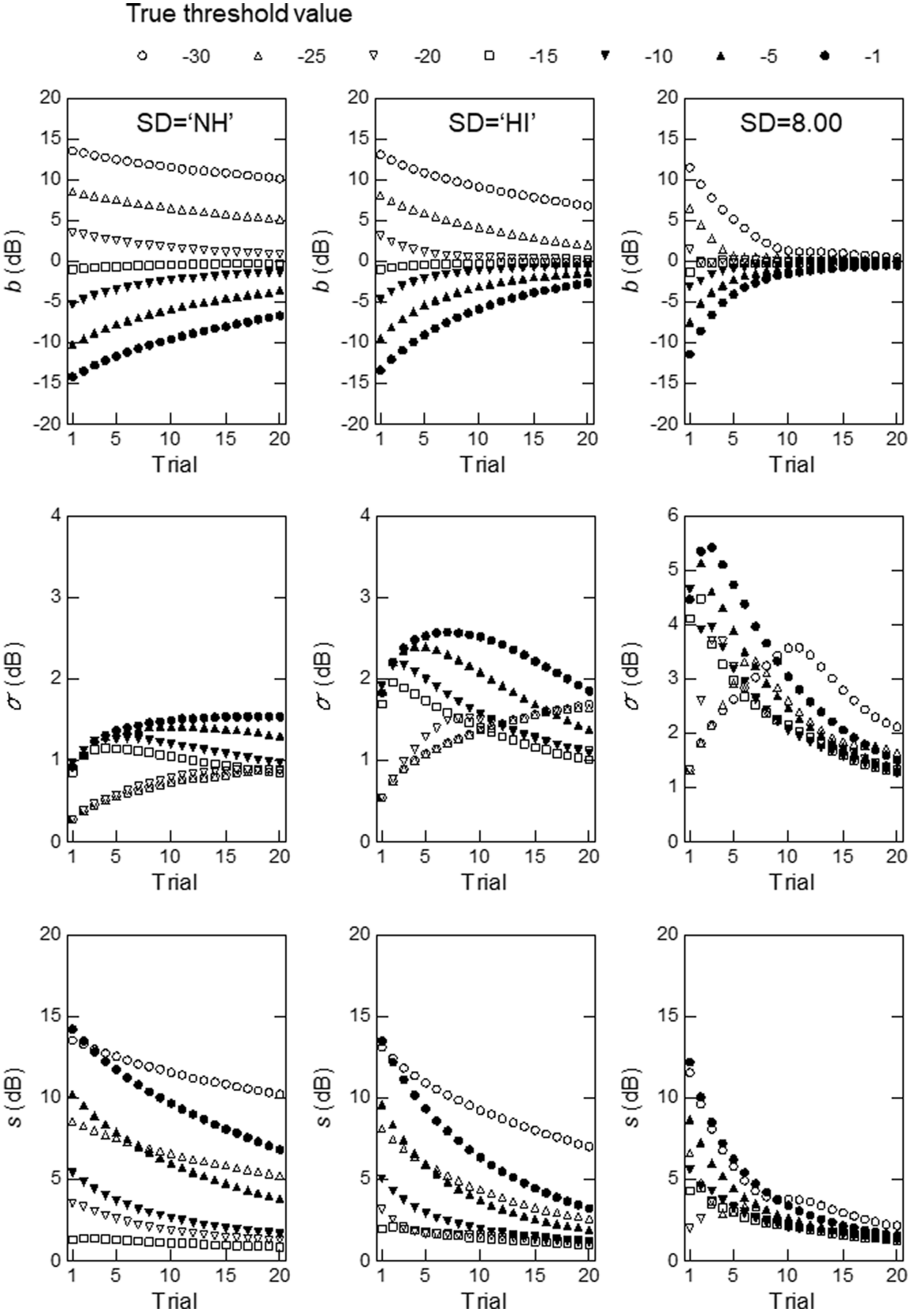
Simulation results obtained with the mean of the thresholds for the PL group and the standard deviation of the thresholds for the NH (left) or HI (middle) group at a modulation frequency of 128 Hz, or a hypothetical standard deviation value of 8.00 (right). The measures used were, from top to bottom, measurement bias, the standard deviation of the threshold estimates, and the total rms error.

When the mean and SD values for the HI group were used (middle), b rapidly approached 0 as the number of trials increased for T = −25, −20, −15 (squares), and − 10 dB (inverted filled triangles). For the other values of T , b changed slowly and did not reach 0 by trial 20, but these changes were larger and occurred faster than those observed for the NH group. When the mean and SD values for the PL group were used (right), the overall pattern was similar to that produced by the mean and SD values for the HI group, although b was further from 0 and changed more slowly than for the HI group at the same values of T .

As shown in the middle row of Figure 4, the mean and SD values for the NH group produced a markedly different pattern of the SD of the estimated thresholds, σ , from those produced by the mean and SD values for the HI and PL groups. For the NH group, the σ values were small, all below 2 dB, and they increased in the first few trials and then leveled off afterward for T = −30 and −1 dB, whereas they gradually decreased for the other values of T . For the HI group, the σ values were much larger, especially in the first 10 trials, and they changed more markedly with the number of trials than those for the NH group at the same values of T . For the PL group, the σ values showed similar patterns to those seen for the HI group, but they were somewhat smaller.

As shown in the bottom row of Figure 4, the rms, s , of the estimated thresholds decreased monotonically as the number of trials increased, with a couple of exceptions (the first few trials for T = −25 dB for the HI and PL groups). The s values were smaller and decreased more rapidly for the HI group than for the NH or PL group, and they appeared to reach the asymptote at around trial 20 for T = −25 to −5 dB. From all these results, the mean and SD values for the HI group seemed to yield higher precision and efficiency at 16 Hz than those for the NH and PL groups.

The results for 128 Hz shown in Figure 5 indicate that the mean and SD values for the PL group led to better overall ZEST performance than those for the NH or HI group. When the parameter values for the PL group were used, b was smaller and converged to 0 more rapidly, especially for T = −30 to −15, than those for the NH and HI groups. The mean and SD values for the PL group produced much larger σ values than those for the NH or HI group. s was smaller and came close to 0 more rapidly for T = −30 to −15 dB for the PL group than for the NH or PL group, although it was small for T = −10 to −1 dB for the HI group. The latter results may reflect the fact that the mean threshold of the HI group, −10.92 dB was closer to the aforementioned T values than that for the PL group, −16.13 dB (see Table 1). Even for T = −10 to −1 dB, σ became small for the PL group as the trial number increased to 20, at a level similar to the values obtained for the HI group.

For other modulation frequencies, we found similar observations. The results for 8 Hz show a similar pattern to that for 16 Hz; i.e., the mean and SD values for the HI group yielded better performance than those for the NH or PL group. For 32, 64, and 256 Hz, we observed that the mean and SD values for the PL group produced better performance than those for the NH or HI group.

The differences observed among the modulation frequencies were probably due to the relative sizes of the SD of the thresholds for the HI and PL groups. At modulation frequencies of 8 and 16 Hz, where the parameter values of the HI group produced better performance, the SD for the HI group was larger than that for the PL group. At the other modulation frequencies, the PL group exhibited larger SD values. The NH group had the smallest SD values at all modulation frequencies. It appears that the size of $\sigma_{0}$ can have an effect on the efficiency and precision of ZEST, which is codified in terms of the measurement bias, b , and the SD of the threshold estimates, σ . For our ZEST method, we used a Gaussian distribution for the initial *pdf*. The posterior *pdf* calculated from Equation 1 in the first and subsequent trials, shifts along a modulation depth (dB) axis, and the size of the shift depends on the size of its variance, which is dependent on the variance (or $\sigma_{0}$ ) of the initial *pdf*. The posterior *pdf* moves over a longer distance when its variance is relatively large; thus, the *pdf* moves farther toward the true value of the threshold, T , resulting in a large decrease in b toward 0 as the number of trials increases. On the other hand, the SD of the threshold estimates, σ , increases as the variance of the posterior *pdf* increases, for the estimates are more variable when the prior *pdf*, which is the posterior *pdf* in the previous trial, has a greater variance.

To confirm the above conjecture regarding the effects of $\sigma_{0}$ on b and σ , we conducted simulations using the mean for the PL group at a 128-Hz modulation frequency (the right column of Figure 5), but replaced the SD value with that for the NH or HI group for 128 Hz, or a hypothetical value of 8.00. Figure 6 shows the results. As can be seen, the graphs for the simulations in which the SD values for the NH (left) or HI (middle column) group were used show very similar patterns to those obtained for the NH and HI groups (Figure 5), except for the vertical positions of the curves, which reflect the differences in the $\mu_{0}$ values used for the simulations. In the right column of graphs, where a very large value of $\sigma_{0}$ (8.00) was used for the simulations, consistent, but extreme patterns are observed. In the top graph, b converges to and nearly reaches 0 rapidly for all values of T as the trial progresses. In the middle graph, σ is much larger and changes more greatly with the number of trials (notice that the scale of the vertical axis has been widened) compared with the patterns obtained with the $\sigma_{0}$ for the NH (left) or HI (middle column) group. In the bottom graph, s is smaller and approaches 0 more rapidly than when the SD for the NH or HI group was used. As mentioned earlier, s is a composite measure of b and σ , and s largely reflects b , which is larger in absolute value than σ .

#### 4.2.2. Effects of β

**Figure 7:**
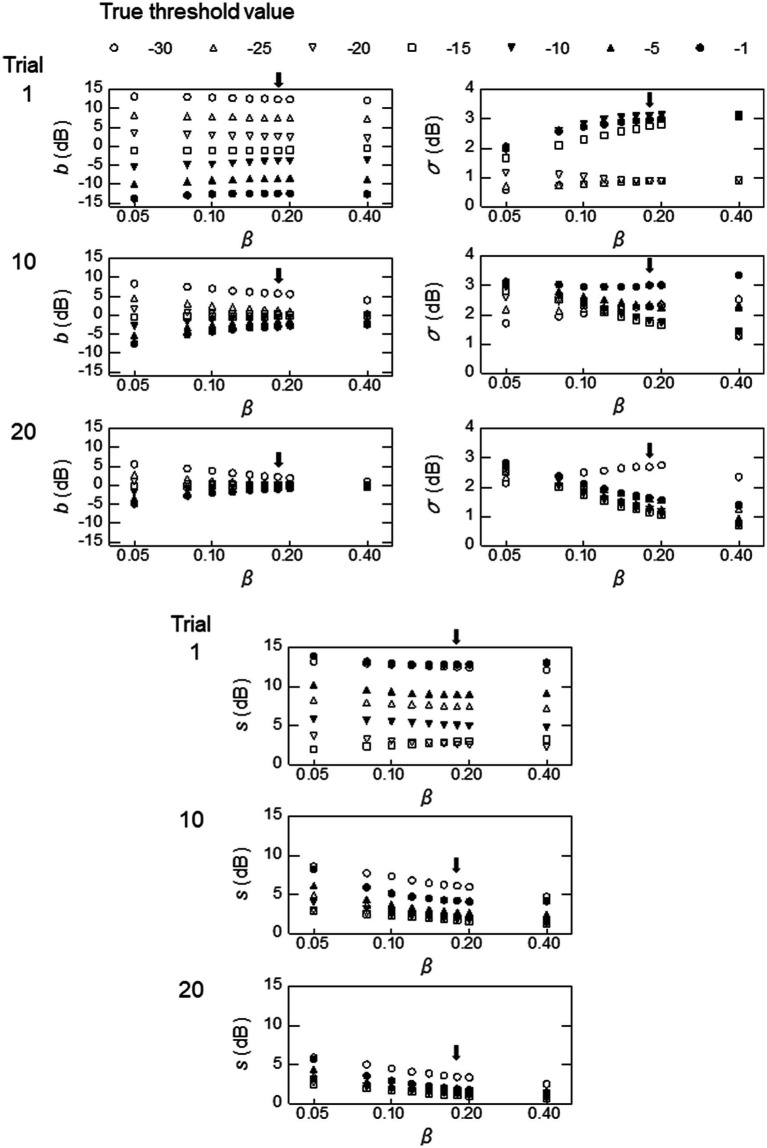
Simulation results obtained with the parameters employed in the PL group at a modulation frequency of 128 Hz, except that the β value was changed from 0.05 to 0.04. The ε value was set at the ideal sweat factor corresponding to the chosen β value. The arrows indicate the results obtained with the same parameter values, including β = 0.18, used for the simulation shown in the left column of Figure 5.

We ran simulations by manipulating the β values from 0.05 to 0.40, which encompassed the values obtained from the empirical psychometric functions in our study (Table 2). To illustrate the effects of β in our simulations, Figure 7 shows the three performance measures, b , σ , and s as a function of β , with the other parameter values set at those for the PL group for 128 Hz (the right column of Figure 5). The value of b (top left column of Figure 7) did not change much with β for each value of T in trial 1. In trial 10, b converged toward 0 as β increased, and by trial 20 they had come close to 0, especially for larger values of β . The σ values (top right column) varied with the value of the true threshold, T , and greater variation was seen with larger β values, except for β = 0.40, at which the variability of σ became somewhat small by trial 20. The s value (bottom column) decreased as the number of trials increased from 1 to 10 to 20, and it was smaller for larger values of β . It should be noted that, although all of these three measures changed with the β value, these changes were less than 2 dB for a given T value when β ranges from 0.10 to 2.0 (the values obtained from the empirical psychometric functions) (Table 2). When we excluded the extreme T values of −30 and −1, the changes with β from 0.10 to 2.0 were within 1 dB. Similar tendencies were observed for the NH and HI groups and for other modulation frequencies.

## 5. Discussion

In this study, we sought to find suitable parameters to use when measuring AM detection thresholds with ZEST. We did this by analyzing the thresholds and the psychometric functions of AM detection. The threshold distributions were quantified using Gaussian distributions while the psychometric functions were fitted using Weibull distributions. These functions (and their fitted parameter values) then served as the initial conditions from which the efficacy of ZEST was explored. In our simulations, we found that the means and SD values of the thresholds for the PL group for the initial *pdf* resulted in better ZEST performance than those for the NH or HI group and that higher β values yielded better simulation performance, but the size of this improvement was relatively small when the β values ranged from 0.1 to 2.0.

These results can be used to set the parameter values of ZEST for real threshold measurements. The following values are appropriate: For the initial *pdf* (Equation 4), $\mu_{0}$ and $\sigma_{0}$ should be set at the mean and SD values of the thresholds for the PL group (Table 1) for each modulation frequency. For the psychometric function (Equation 1), γ = 0.33; δ = 0.02; β should be some value between 0.1 and 0.2 (Table 2), preferably a relatively high value like 0.17, which corresponds to the average of the values reported in Table 2; and ε should be set at the ideal sweat factor corresponding to the chosen value of β . Currently, we are collecting AM detection thresholds using ZEST with the suggested parameter values, together with measurements of gap detection thresholds. The results obtained so far from a total of over 70 participants (NH or HI) are promising, in that the threshold estimates reached an asymptote after 10 to 20 trials, which takes less than 2 min.

While all efforts were made to find sets of parameters that result in efficient and reliable threshold estimation using ZEST, our study had several limitations, which could benefit from further investigation. We used a rather small number of participants for the measurement of psychometric functions. We did so because these measurements were time-consuming (500 trials for each modulation frequency), and we wanted to have an acceptable range of initial parameters to use for the computer simulations, which in turn revealed appropriate parameter values for ZEST. We do not claim that our results capture all of the features of the psychometric functions for AM detection. For example, the psychometric functions obtained from our participants (Figure 3) showed similar shapes and were fitted well by Weibull distributions, although the estimates of β varied among the three participants. Eddins (1993) showed that the slope of psychometric function became steeper with increasing modulation frequency, but our results did not show any such tendency.

A more precise approximation of the initial *pdf* and the distribution of AM detection thresholds is also needed. We assumed that the initial *pdf* can be well described by Gaussian distributions. However, as the modulation frequency increased, the histograms of the NH and HI groups separated, and the prior distribution became bimodal. A better way to approximate the initial *pdf* for the threshold distributions would be to follow Turpin et al. (2003; also see Vingrys and Pianta, 1999) by combining the histograms for all modulation frequencies for the NH and HI groups separately and pooling the combined histograms of the NH and HI groups in a certain ratio. These issues will be explored once a good amount of threshold data has been obtained in our ongoing experiment with human participants.

## Data availability statement

The raw data supporting the conclusions of this article will be made available by the authors, without undue reservation.

## Ethics statement

The studies involving human participants were reviewed and approved by the Research Ethics Board of the Faculty of Information Science and Electrical Engineering, Kyushu University. The patients/participants provided their written informed consent to participate in this study.

## Author contributions

TM organized the database of AM thresholds. SM conducted the measurements of psychometric functions and wrote the first draft of the manuscript. SM and YM performed the simulations. All authors contributed to the conception, design of the study, manuscript revision, read, and approved the submitted version.

## Funding

This research was supported by a Grant-in-Aid from the Japan Society for the Promotion of Science for Challenging Research (Exploratory) 20K2066 and the Soda Toyoji Memorial Foundation to SM.

## Conflict of interest

TM was employed by RION Co., Ltd.

The remaining authors declare that the research was conducted in the absence of any commercial or financial relationships that could be construed as a potential conflict of interest.

## Publisher’s note

All claims expressed in this article are solely those of the authors and do not necessarily represent those of their affiliated organizations, or those of the publisher, the editors and the reviewers. Any product that may be evaluated in this article, or claim that may be made by its manufacturer, is not guaranteed or endorsed by the publisher.

## Footnotes

^1^While Morimoto et al. (2018) were able to obtain detection thresholds for all of the listeners with HI at *f*_m_ of 8 Hz, some of those listeners were unable to complete the task at the higher *f*_m_, so that the total numbers can differ from the total of 39 participants. The exact numbers of the participants are reported in the legend of Figure 2.

## Abbreviations

AM: amplitude modulation
TMTF: temporal modulation transfer function
*f*_m_: modulation frequency
*S*_p_: peak sensitivity
*f*_c_: cut-off frequency
*pdf*: probability density function
NH: normal hearing
HI: hearing impaired
