# Supplementary material for: Tests of human auditory temporal resolution: preliminary investigation of ZEST parameters for amplitude modulation detection
Source: Front Neurosci. 2023 Jul 5;17:1148476. doi: 10.3389/fnins.2023.1148476 (PMC10354262; doi:10.3389/fnins.2023.1148476)
Supplement: Supplementary file 1 [file Presentation_1.pptx]

## Slide 1
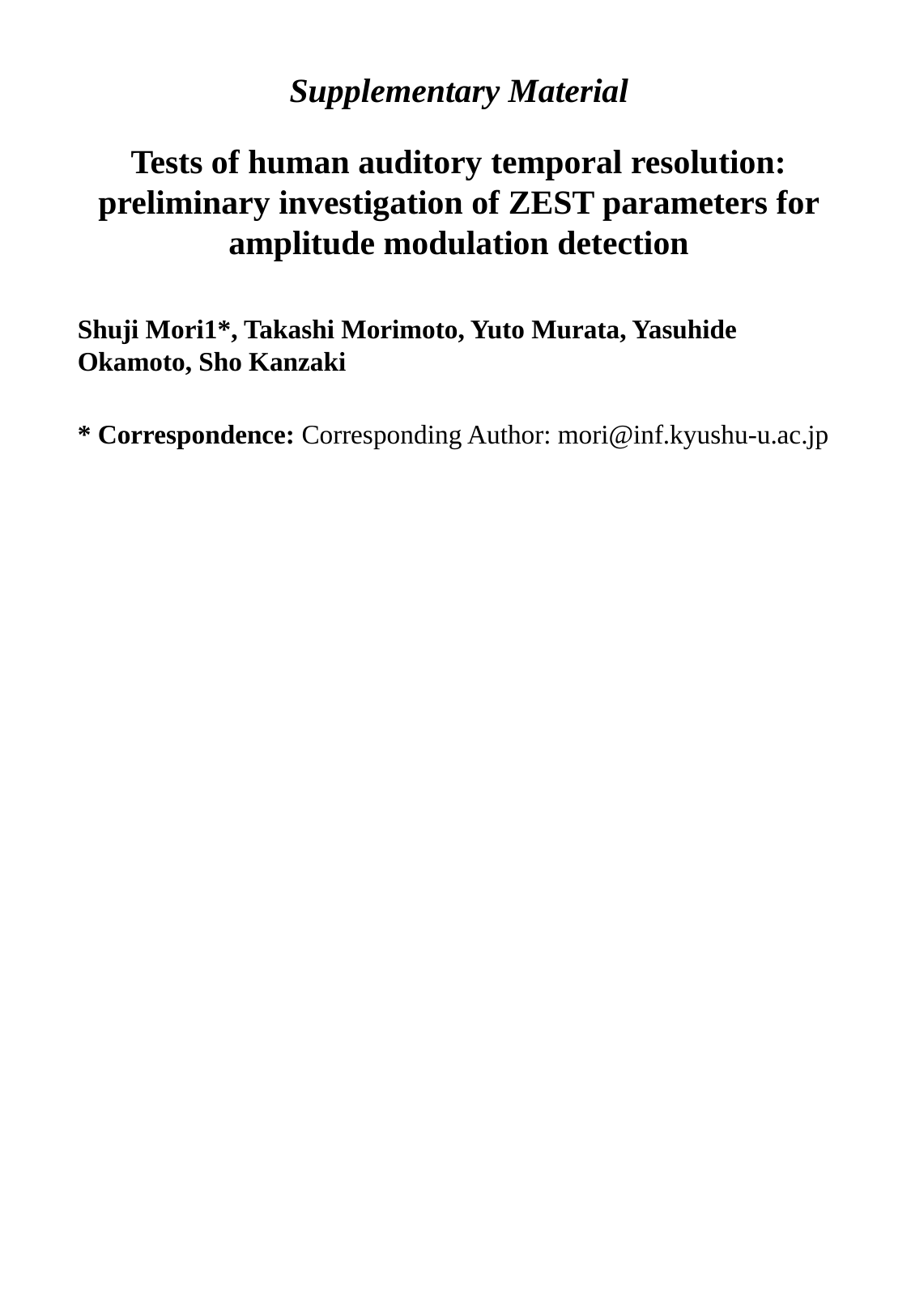

Supplementary Material
Tests of human auditory temporal resolution: preliminary investigation of ZEST parameters for amplitude modulation detection
Shuji Mori1*, Takashi Morimoto, Yuto Murata, Yasuhide Okamoto, Sho Kanzaki
* Correspondence: Corresponding Author: mori@inf.kyushu-u.ac.jp

## Slide 2
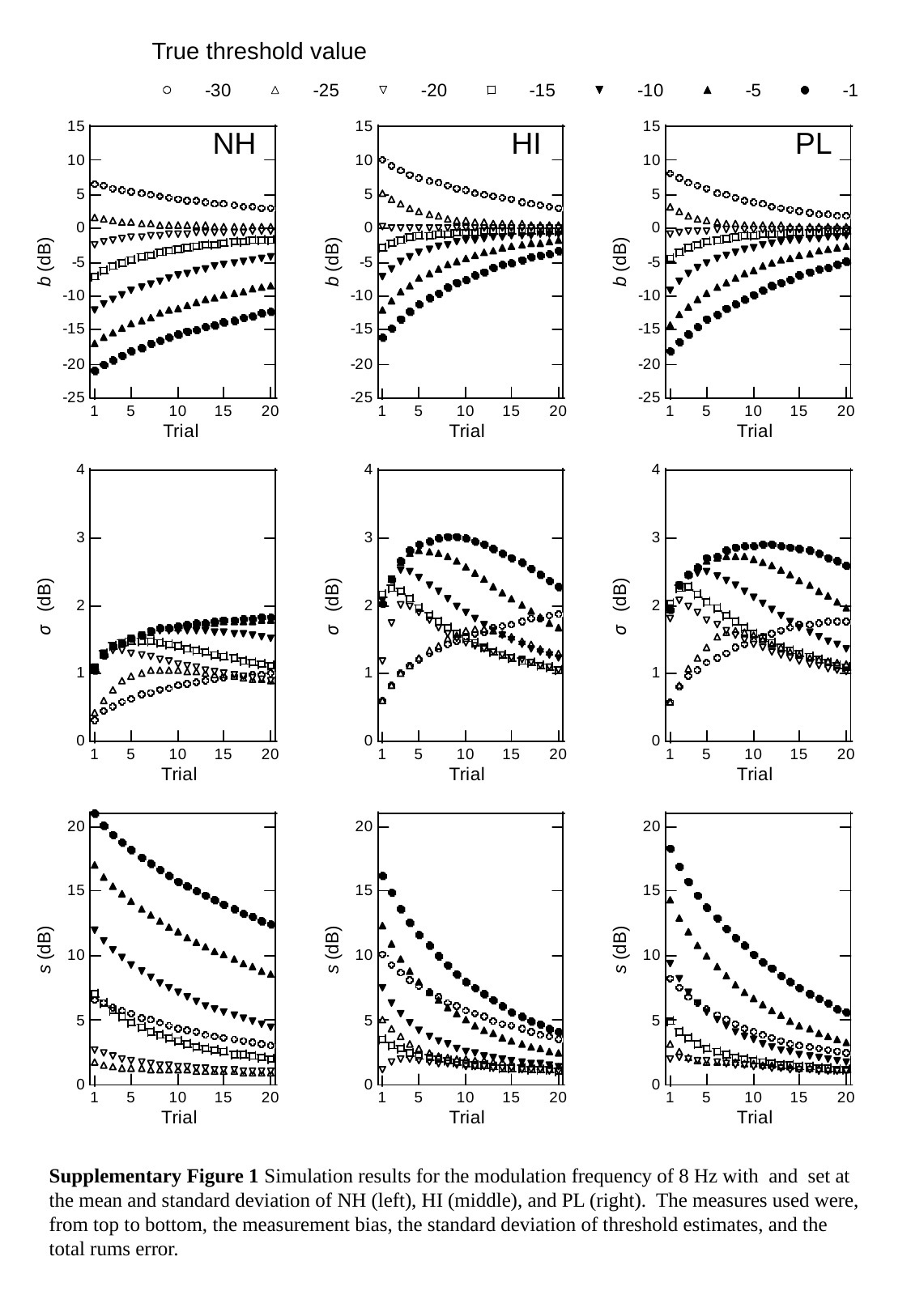

True threshold value
NH
HI
PL

## Slide 3
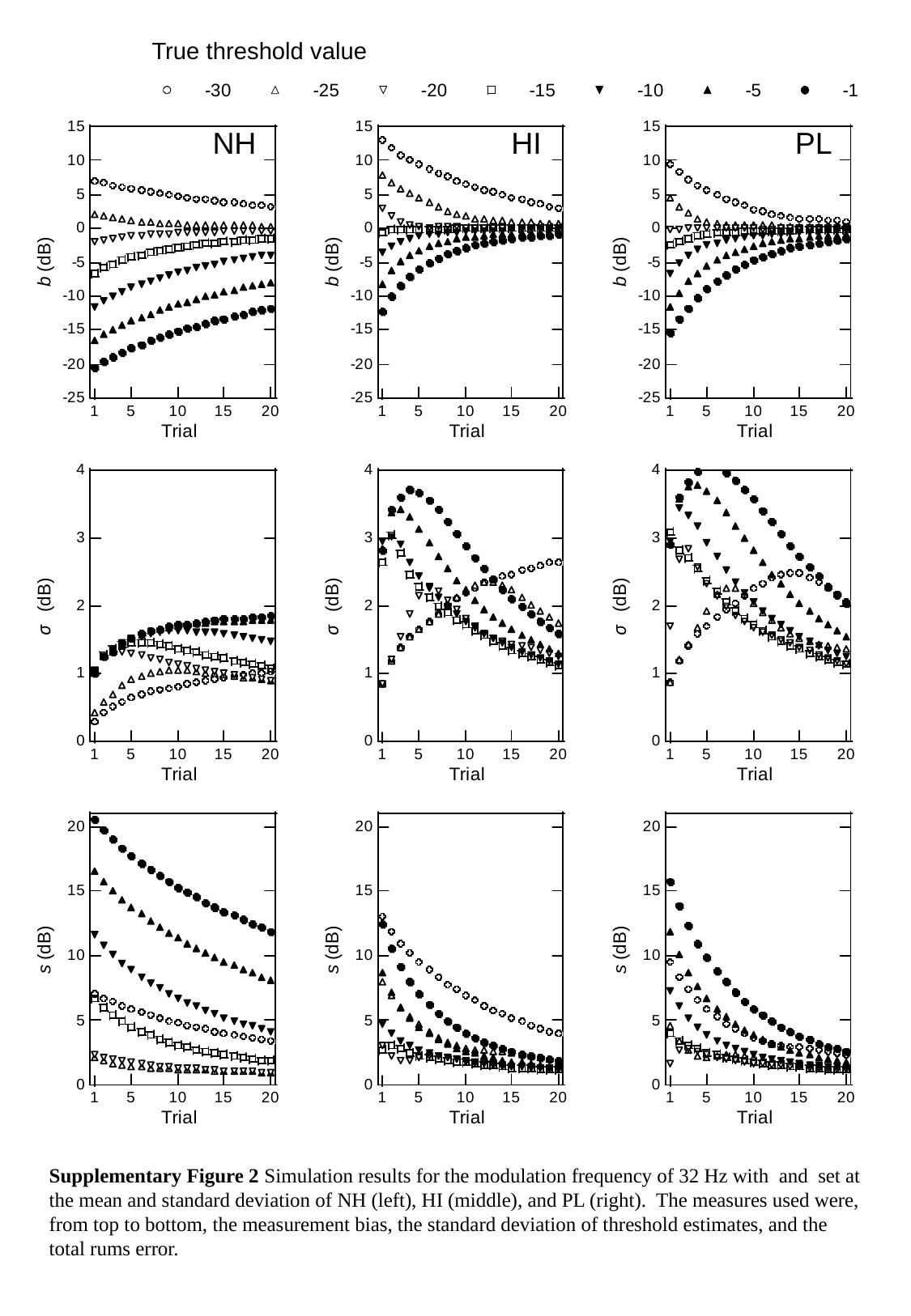

True threshold value
NH
HI
PL

## Slide 4
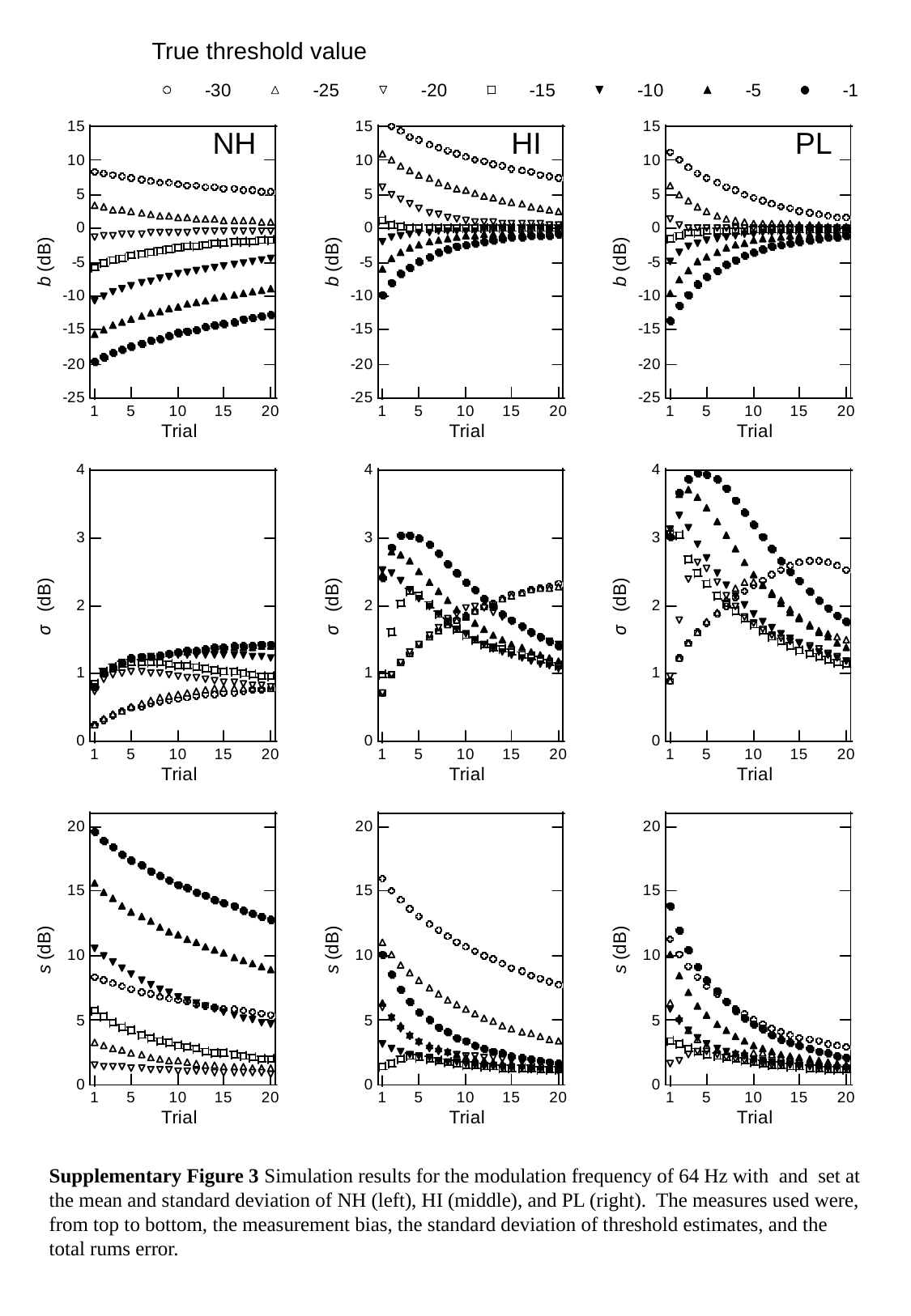

True threshold value
NH
HI
PL
